# Tubulin couples death receptor 5 to regulate apoptosis

**DOI:** 10.18632/oncotarget.26407

**Published:** 2018-12-04

**Authors:** Julianne D. Twomey, Liqun Zhao, Shen Luo, Qing Xu, Baolin Zhang

**Affiliations:** ^1^ Office of Biotechnology Products, Center for Drug Evaluation and Research, Food and Drug Administration, Silver Spring, MD 20993, USA

**Keywords:** tubulin, death receptor 5, TRAIL, apoptosis, cancer therapy

## Abstract

Activation of death receptor 5 (DR5) to induce apoptosis in cancer cells is an attractive strategy for cancer therapy. However, many tumor cell lines and primary tumors are resistant to DR5 targeted agents including recombinant tumor necrosis factor (TNF)-related apoptosis-inducing ligand (TRAIL) and anti-DR5 agonistic antibodies. Here we identify tubulin proteins - primarily consisting of α and β subunits folded into microtubule polymers - as a crucial modulator of DR5 mediated apoptosis. Using affinity purification coupled with mass spectrometry, we found that DR5 interacts with both α- and β-tubulin proteins in cancer cells. Pharmacological disruption of microtubules increased DR5 protein expression and subsequently sensitized the cells to TRAIL-induced apoptosis. Similar results were observed by selectively silencing tubulin transcript using small RNA interference. We also demonstrate that tubulin/microtubule blockade augments TRAIL induced apoptosis by stabilizing DR5 protein. Together, our results link the tubulin/microtubule network to the stringent regulation of DR5 mediated apoptosis, which could lead to potential therapeutic strategies to enhance cancer therapy efficacy.

## INTRODUCTION

Death receptor-5 (DR5), also known as TRAIL receptor 2 (TRAIL-R2), is a cell surface receptor of the TNF-receptor superfamily that contains a cytoplasmic death domain [1]. This receptor transduces apoptotic signals from its physiological ligand – tumor necrosis factor-related apoptosis inducing ligand (TRAIL) – or its agonistic antibodies [2]. Upon activation, DR5 clusters into homotrimers to assemble the adapter protein Fas-associated death domain protein (FADD) and procaspase 8 or 10 into a death-inducing signaling complex (DISC), leading to activation of the caspase cascade and apoptotic execution in targeted cells [3]. The DR5/TRAIL receptor-ligand system has been implicated in immunosurveillance of tumor cells [4]. Therefore, it serves as an attractive therapeutic target for cancer treatment. Over the past decade, multiple clinical trials have been initiated to test the potential antitumor activities of recombinant human TRAIL (rhTRAIL) and anti-DR5 agonistic antibodies [5, 6]. Unfortunately, many tumor cell lines and primary tumors were found to be resistant to those agents. The underlying mechanisms remain incompletely defined; although the deficiency in DR5 itself does play a role in rendering cancer resistance to DR5 targeted therapy [7-11]. In this regard, DR5 has been shown to undergo rapid internalization in a ligand-dependent manner [8, 12] and sequester into intracellular compartments such as the nucleus [9] or autophagosome vesicles [10], leading to its absence on the surface membrane of targeted cells.

In a general scheme, apoptotic cell death is characterized by distinct morphological and biochemical changes: cell shrinkage, plasma membrane “blebbing”, chromatin condensation, and DNA and cells fragmentation [11]. These dramatic structural changes proceed with a series of profound alterations in the cell cytoskeleton components, including assembly and disassembly of microtubule networks. Microtubules are highly dynamic, hollow, cylindrical structures formed by α-tubulin and β-tubulin heterodimers [13]. In response to apoptosis induction, the tubulin/microtubule structures, which are initially depolymerized, repolymerize to form an apoptotic microtubule network (AMN) beneath the plasma membrane. The dynamic changes in microtubules assist in the dispersal of nuclear and cellular fragments and may help to preserve the integrity of the plasma membrane of the dying cell [14]. Therapeutic agents targeting microtubules, including stabilizing (e.g. docetaxel, epothilione) or destabilizing (e.g., vincristine, colchicine) agents, induce apoptosis in targeted cells and therefore are widely used for treating solid tumors and hematopoietic malignancies [15]. Recent evidence shows that tubulin depolymerizing agents (e.g., paclitaxel) synergize with TRAIL to kill cancer cells [1, 16-19]. In this study, we demonstrate that tubulin interacts with DR5, leading to DR5 protein degradation in cancer cells. Blockade of tubulin/microtubules stabilizes DR5 expression on the cell surface and enhances TRAIL-induced apoptosis. Tubulin-stabilizing agents also increased DR5 expression and TRAIL-sensitivity. These data provide a rational for combinational strategies using tubulin/microtubule and DR5 targeted agents in cancer treatment.

## RESULTS

### Tubulin interacts with death receptor 5

**Figure 1:**
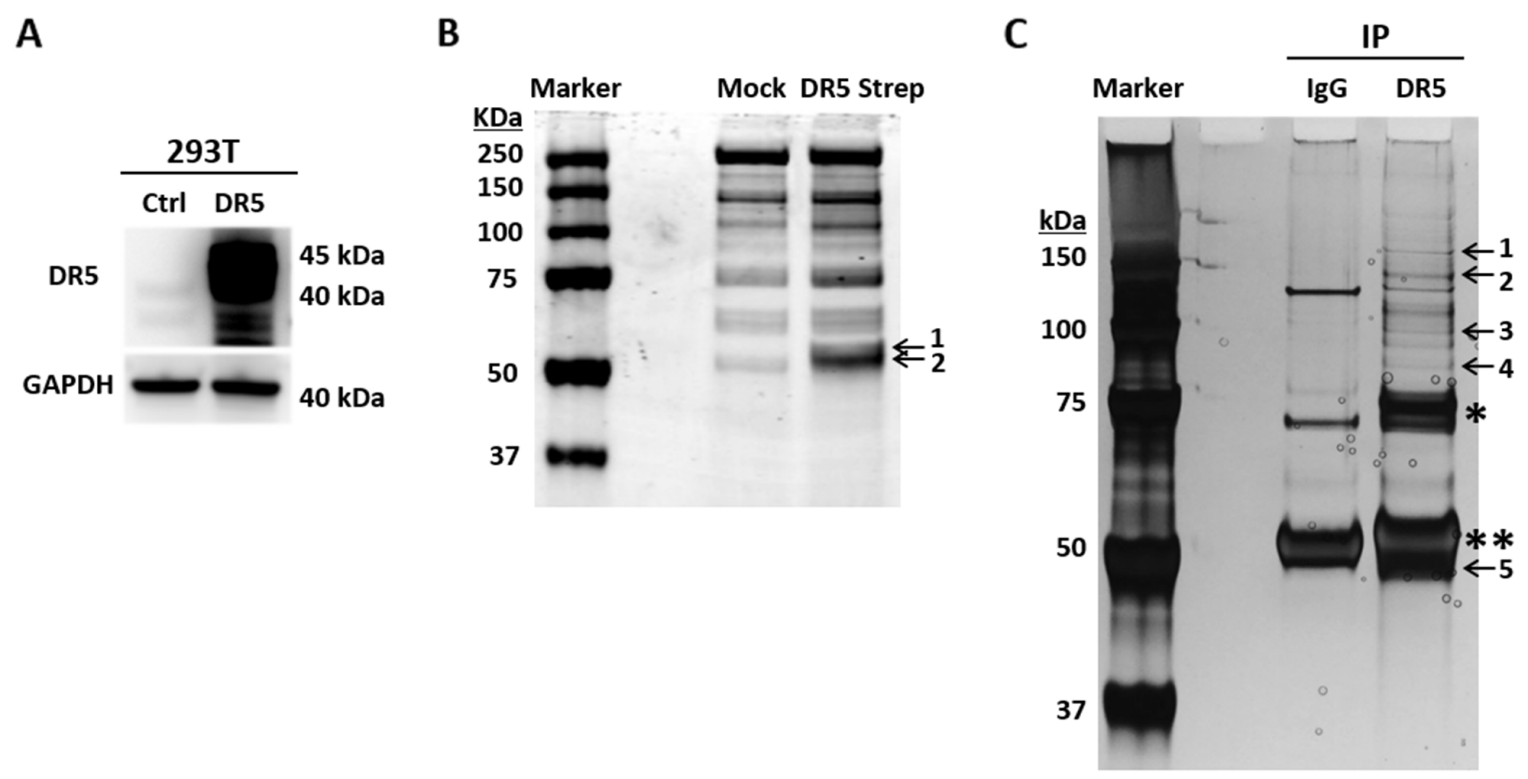
Affinity purification of DR5-binding proteins **(A)** 293T cells were transfected with a cDNA plasmid encoding Strep-DR5 fusion protein or an empty vector as a control. Shown are representative DR5 immunoblots. **(B)** Strep-DR5 protein complexes were isolated using Strep-Tactin affinity chromatography and resolved onto SDS-PAGE followed by Coomassie blue staining. Labeled bands were identified by mass spectrometry as tubulin (band 1) and DR5 (band 2). **(C)** Anti-DR5 immunoprecipitation followed by SDS-PAGE and silver staining. Labeled bands are as follows: 1, Polyubiquitin-B/C; 2, Isoleucine-tRNA ligase; 3, Exportin-2; 4, HSP 90-beta; 5, Tubulin & DR5. ^*^unknown component in the commercial anti-DR5 antibody, ^**^IgG heavy chain.

**Figure 2:**
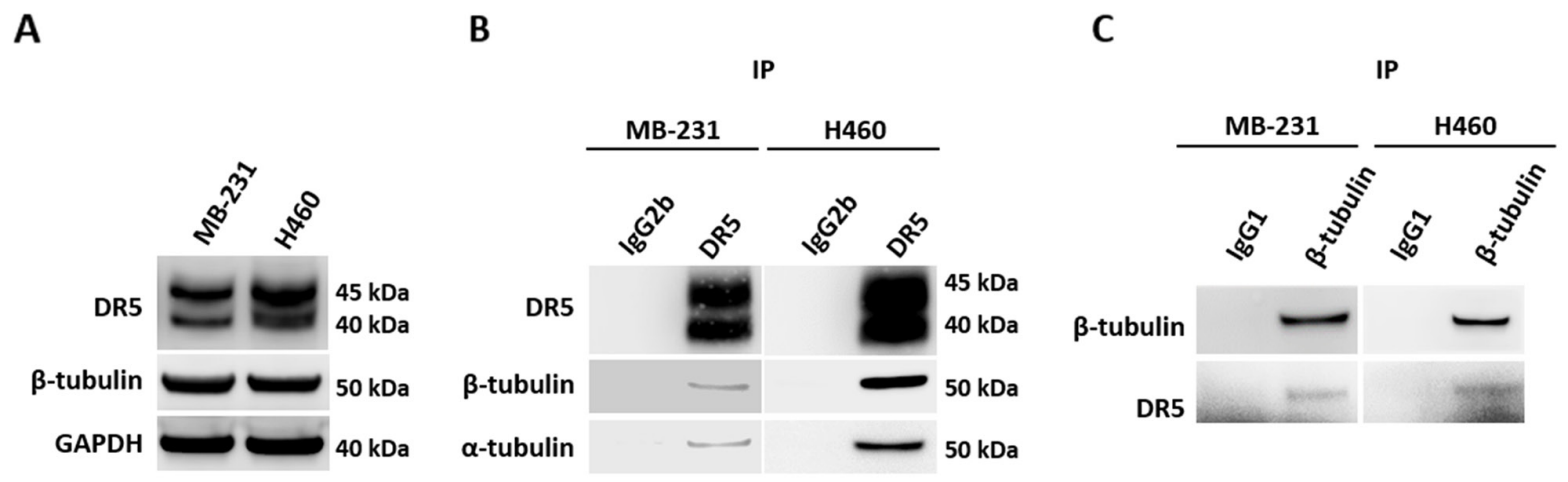
Endogenous DR5 and tubulin interaction **(A)** Immunoblots of endogenous DR5 and β-tubulin proteins in MB-231 and H460 cell lines. **(B)** Cell lysates were subjected to IP with anti-DR5 antibodies or IgG1 or IgG2b control**s**. IP samples were immunoblotted as indicated. **(C)** Reverse co-IP using anti-tubulin antibodies confirmed the interaction between tubulin and DR5.

To identify DR5 interacting proteins, we transfected 293T cells with a cDNA plasmid expressing Strep-tagged DR5 fusion protein or an empty plasmid (Figure 1A). Strep-DR5 protein complexes were purified using Strep-Tactin affinity chromatography. The purified proteins displayed distinct protein bands at ∼ 50 kDa on SDS-PAGE, which were barely detectable in the samples prepared from cells transfected with an empty plasmid (Figure 1B). Using mass spectrometry, we identified tubulin and DR5 in the protein bands. In parallel, we performed co-immunoprecipitation (co-IP) of Strep-DR5 protein complexes using an anti-DR5 monoclonal antibody. Both tubulin and DR5 were present in the anti-DR5 immunocomplexes (Figure 1C). Of note, several other proteins were also detected in anti-DR5 immunocomplexes; including exportin-2, transportin and Heat shock protein 90 (HSP90). Whether these proteins are involved in tubulin-DR5 interaction and their potential roles in DR5 functions remains to be determined. Given the known functions of tubulins in apoptosis, we focused on characterization of tubulin-DR5 interaction. We then performed co-IP experiments to isolate endogenous DR5 immunocomplexes using anti-DR5 and anti-tubulin antibodies, respectively. Stable DR5-tubulin complexes were detected in both MB-231 and H460 cell lines (Figure 2). These results demonstrate that tubulin physically interacts with DR5 in cancer cells.

### Tubulin negatively regulates DR5-mediated apoptosis

**Figure 3:**
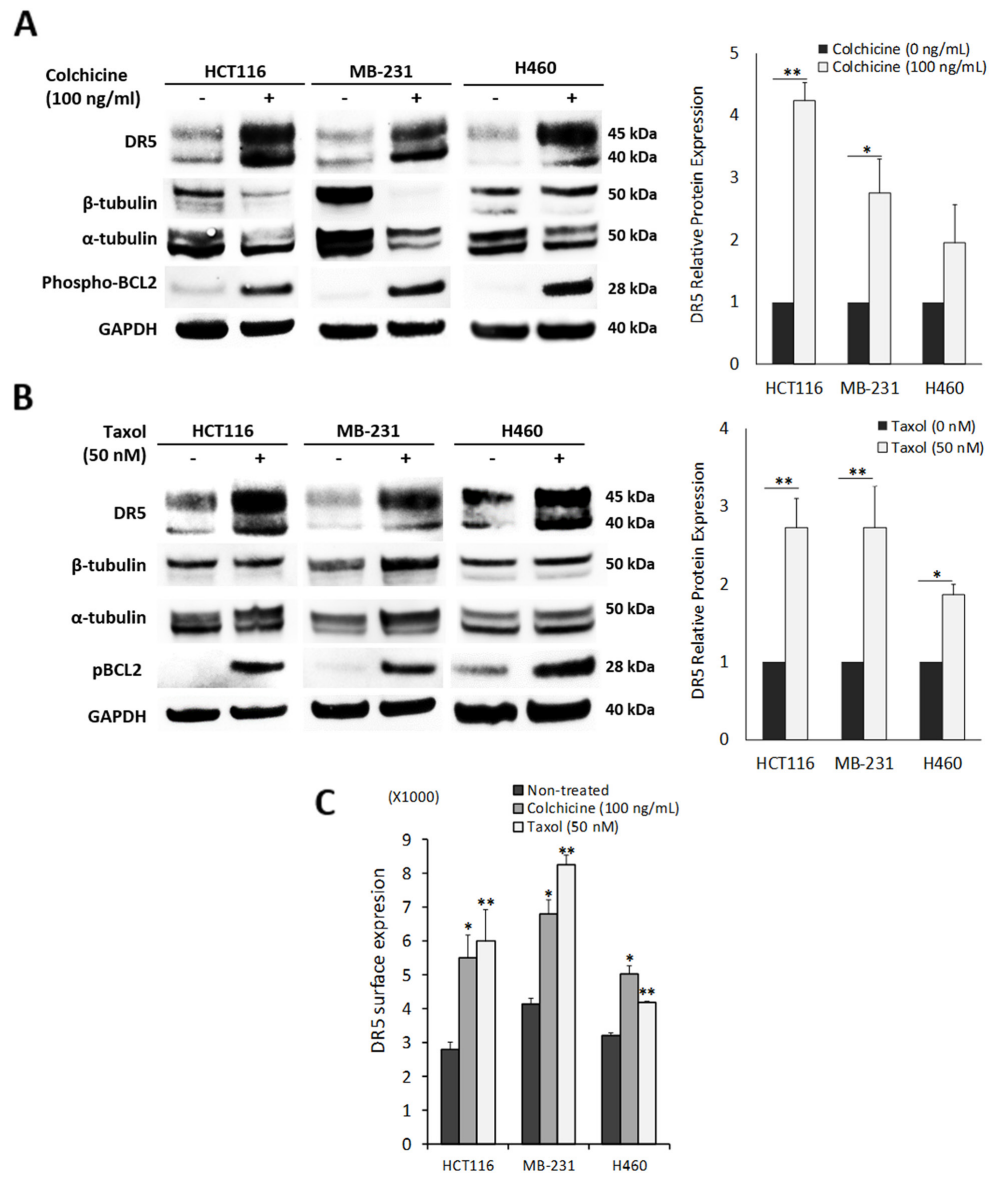
Pharmacological disruption of tubulin increases DR5 expression **(A)** Cells were treated with colchicine (tubulin inhibitor) at 100 ng/ml for or **(B)** taxol (tubulin stabilizer) at 50 nM 16 h, and analyzed by immunoblotting or **(C)** flow cytometry. DR5 total expression is relative to untreated controls. Values are means ± SD of triplicates, ^*^p<0.05, ^**^p<0.01, N=3.

**Figure 4:**
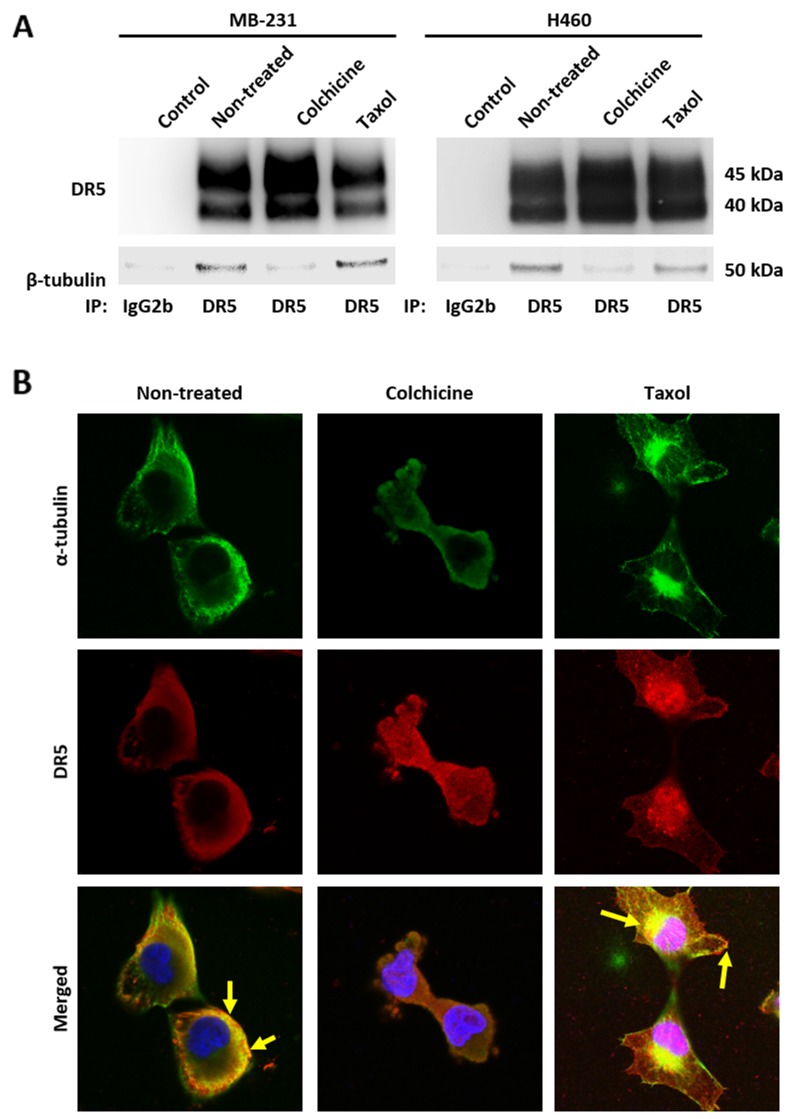
Disruption of tubulin assembly decreases affinity to DR5 **(A)** Cells were treated with colchicine at 100 ng/mL or taxol at 50 nM for 16 hours and then cell lysates were subjected to IP with anti-DR5 antibodies or IgG2b control. **(B)** MB-231 cells were treated with colchicine at 50 ng/mL or taxol at 25 nM for 4 hours and stained for α-tubulin (green) and DR5 (red) with DAPI co-staining. Cells were visualized using confocal microscopy. Yellow arrows indicate co-localization of α-tubulin and DR5. Images were taken at 40x magnification.

**Figure 5:**
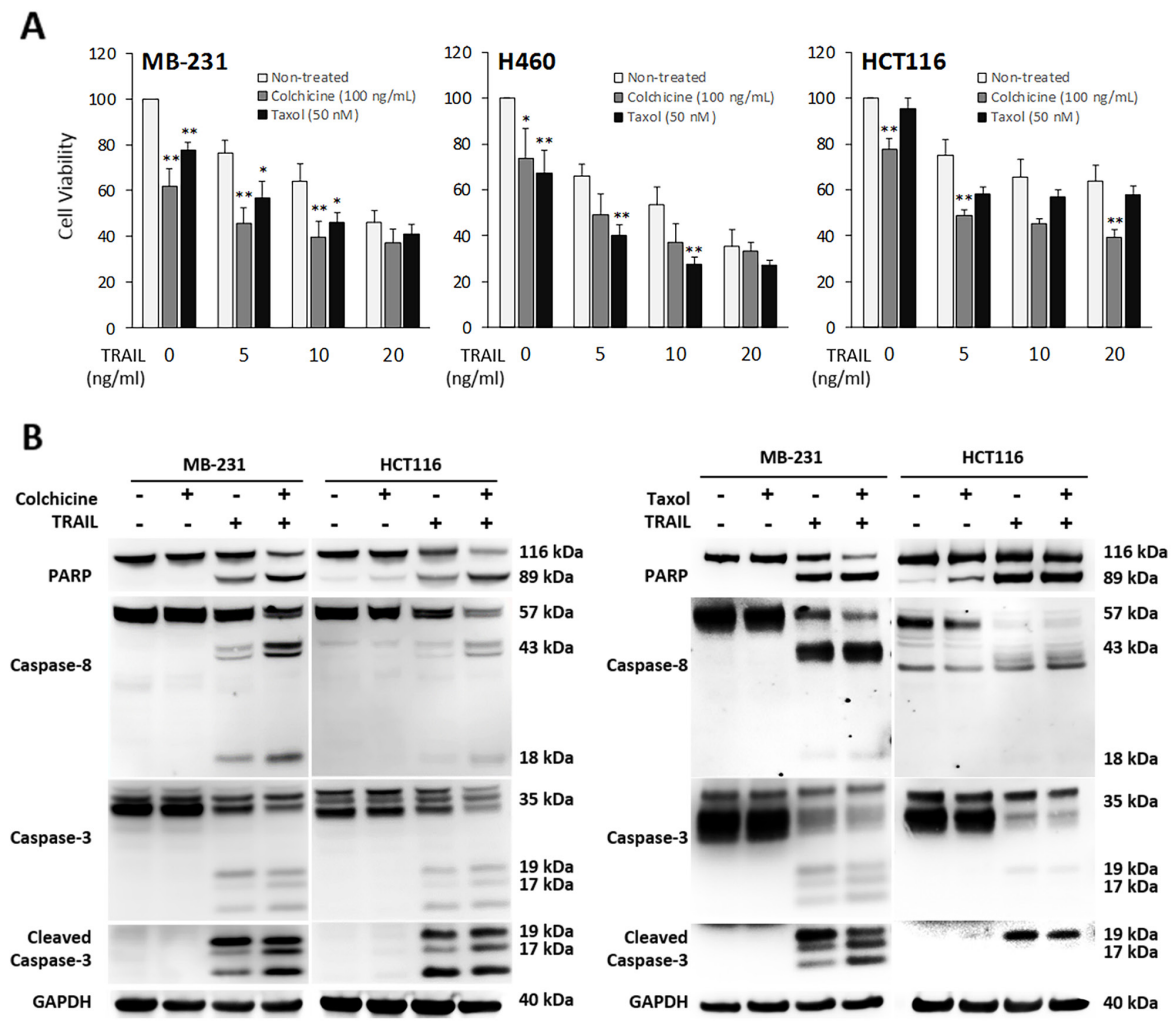
Pharmacological inhibition of tubulin enhances TRAIL-induced apoptosis Cells were treated with colchicine at 100 ng/ml or taxol at 50 nM in combination with TRAIL at the indicated doses for 16 h. The resulting cells were analyzed for cell viability **(A)** and caspase activation (TRAIL at 10 ng/mL) **(B)**. Values are means ± SD of triplicates, ^*^p<0.05, ^**^p<0.01, N=3.

**Figure 6:**
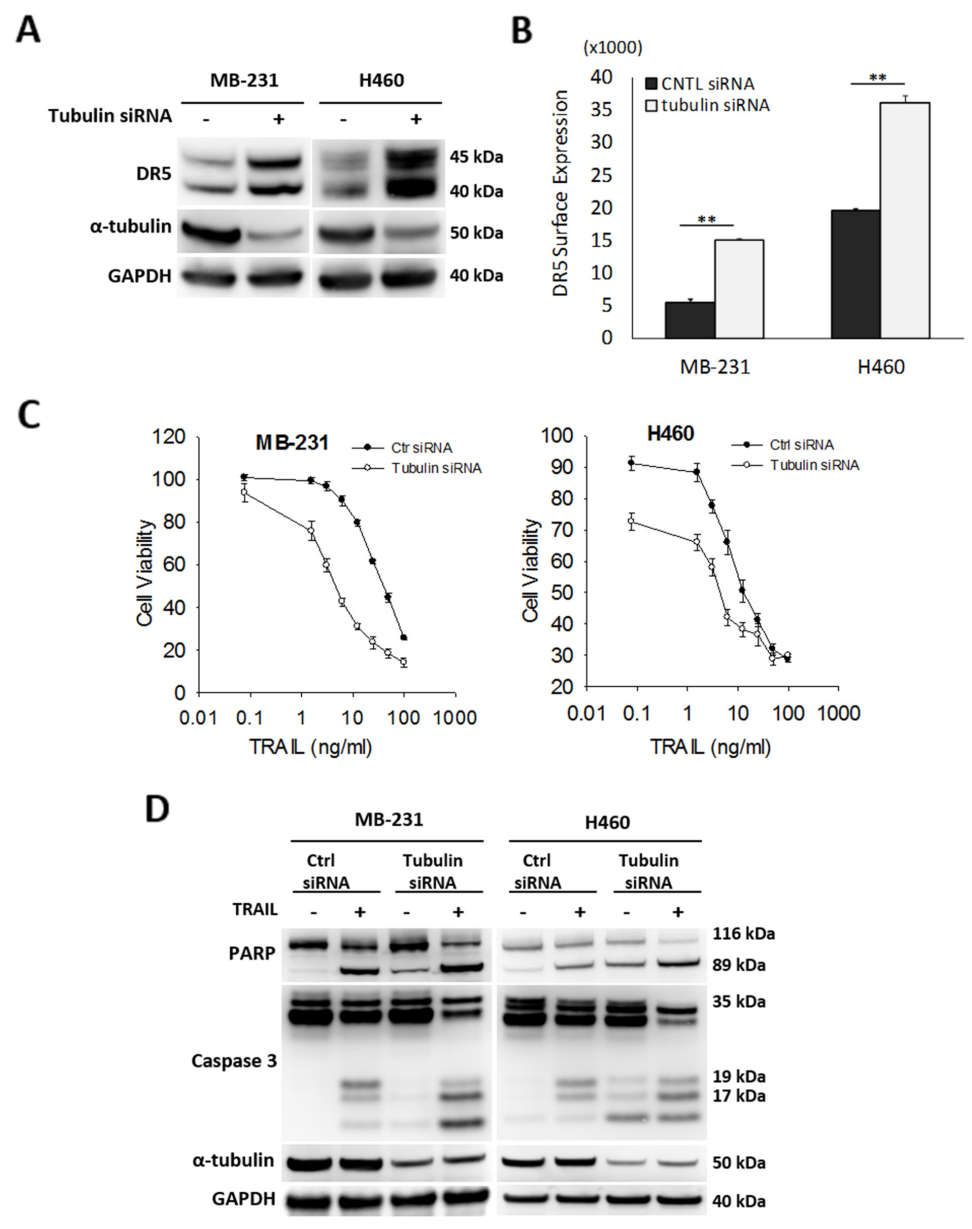
Silencing tubulin increases DR5 expression and TRAIL induced apoptosis **(A)** Cells were transfected with control siRNA (-) or siRNA against α-tubulin (+) for 48 hr, followed by immunoblot analysis. **(B)** Relative levels of cell surface DR5 was determined by flow cytometry. Cells were prepared as in (A) and stained with PE-conjugated monoclonal anti-DR5 antibody or isotype-matched control IgG (IgG2b). Data shown is relative to IgG2b-PE isotype controls. Values are means ± SD, ^**^p<0.01, N=3. **(C & D)** Cells were transfected with siRNA as in (A), treated with TRAIL at 10 ng/ml for 24 h, followed by analyses for cell viability (C) and caspase activation (D).

Given the stable tubulin-DR5 complex formation, we tested the role of tubulin in regulating TRAIL induced apoptosis by manipulating cellular tubulin polymerization. To this end, we treated the cells with colchicine – a microtubule inhibitor that is known to selectively bind tubulin and disrupt microtubule polymerization or taxol- a microtubule stabilizer which inhibits the disassembly of microtubules [20, 21]. Notably, both colchicine and taxol potently upregulated DR5 protein levels in all the cell lines tested (MB-231, H460 and HCT116) (Figure 3A & 3B). In colchicine treated cells, the inhibition of microtubule assembly resulted in a decrease in total α and β tubulin expression and disruption of the DR5-tubulin complexes (Figure 4A, Supplementary Figure 1). This was accompanied by in an increased localization of DR5 throughout the cytoplasm and to the cell surface (Figure 4A & 4B). Treatment with taxol (stabilizing the tubulin network) had little or no effect on tubulin-DR5 interactions or the expression levels of tubulin monomers (Figure 3B, 4A, & Supplementary Figure 1). However, there was also a considerable increase in DR5 total and surface expression. For both the colchicine and the taxol treatment, the resultant cells were sensitized to TRAIL-induced cell death as demonstrated by cell viability and caspase activation assays (Figure 5A & 5B). The cell viability data were analyzed using CompuSyn software to generate combination index (CI) values for each treatment condition (Table 1). The results (CI values) showed a synergistic effect of colchicine and TRAIL. Similar observations were made when tubulin was specifically silenced by small RNA interference (siRNA) (Figure 6). Knockdown of α-tubulin increased DR5 total protein (Figure 6A) and surface levels (Figure 6B). Consistent with this data, tubulin-deficient cells underwent massive apoptosis following TRAIL treatment (Figure 6C & 6D). Interestingly, silencing tubulin alone (without adding TRAIL) was effective to induce apoptosis in H460 cells (Figure 6D). This effect agrees with previous reports that DR5, when overexpressed on cell surface, can engage self-clustering to trigger spontaneous apoptosis in a ligand-independent mode [22]. Nonetheless, these data demonstrate that blockade or stabilization of microtubule polymer assembly effectively increased DR5 protein expression leading to apoptosis execution. In a cellular context, tubulin appears to regulate apoptosis via DR5.

**Table 1:** Colchicine demonstrates the synergy effect with TRAIL to inhibit cancer cell growth

Cells	TRAIL (ng/ml)	Colchicine (ng/ml)	CI	Description
MB-231	5	100	0.230	Synergism
	10	100	0.341	Synergism
	20	100	0.538	Synergism
HCT116	1.25	100	0.099	Synergism
	2.5	100	0.173	Synergism
	5.0	100	0.297	Synergism
H460	5	100	0.618	Synergism
	10	100	0.578	Synergism
	20	100	0.533	Synergism

### Blockade of tubulin stabilizes DR5 protein

**Figure 7:**
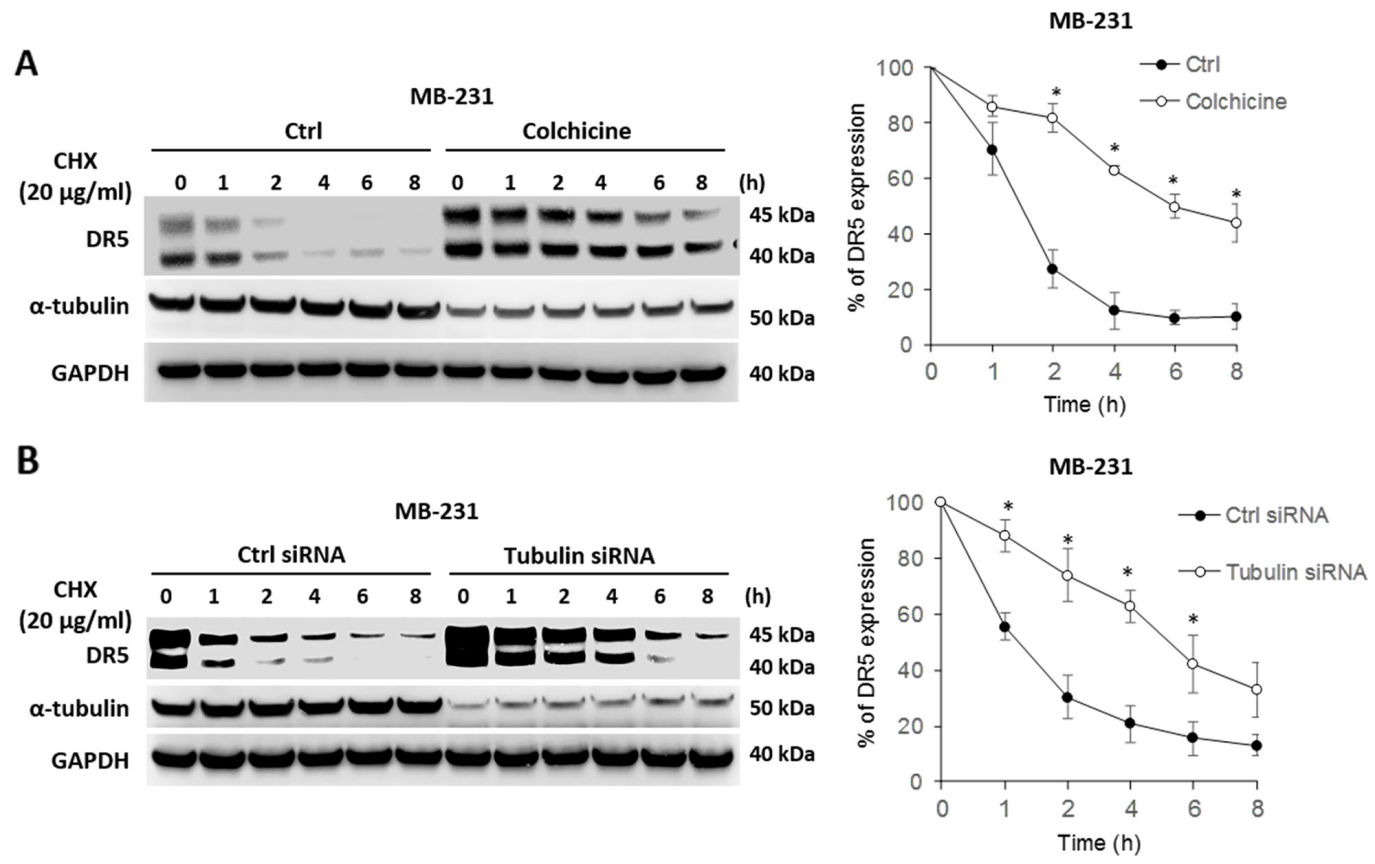
Blockade of tubulin stabilizes DR5 protein **(A)** MDA-MB-231 cells were left untreated or treated with 500 ng/ml of colchicine followed by stimulation with 20 μg/ml of cycloheximide (CHX; a protein synthesis inhibitor) for the indicated times. Right panel, relative DR5 protein levels were quantified from the blots of DR5 and corresponding GAPDH in each sample. **(B)** MDA-MB-231 cells were transfected with control siRNA or siRNA against tubulin for 48 h, and were then treated with 20 μg/ml of CHX for the indicated times, and analyzed as in A. Values are means ± SD, ^*^p<0.05, N=3.

We tested whether tubulin plays a role in regulating DR5 protein stability. To this end, cells were left untreated or treated with colchicine to disrupt tubulin function, followed by treatment with cycloheximide (CHX) – a protein synthesis inhibitor that is widely used to shut down protein synthesis machinery in targeted cells. The levels of pre-existing cellular DR5 protein in MB-231 cells were monitored by immunoblotting at the indicated time points (Figure 7A). DR5 was found to undergo time-dependent degradation under both experimental conditions. Notably, DR5 degradation was significantly delayed in tubulin-deficient cells compared with untreated counterparts. The half-life of DR5 protein in tubulin-deficient cells (∼5-6 hrs) was about 3-4 times longer than that of control cells (1.5-2 hrs). Similar results were obtained when tubulin was silenced in the same cell line (Figure 7B). These data support the notion that cellular tubulin-DR5 complex formation may stimulate DR5 protein degradation. Selective inhibition of tubulin appeared to effectively sensitize cancer cells to DR5 mediated apoptosis; this effect is possibly mediated by stabilizing DR5 protein in targeted cells.

## DISCUSSION

Despite significant progress in understanding how DR5 triggers apoptosis, the molecular mechanisms of tumor resistance to DR5-targeted therapies remain incompletely defined. DR5 is subject to post-translational processing, and its proper expression on the cell surface is a prerequisite for ligand binding and caspase activation [23]. Here we identified tubulin as a functional regulator of DR5-mediated apoptosis. By forming stable complexes, tubulin appears to negatively regulate DR5 protein stability thereby limiting its apoptotic potential. Our findings uncover an important link between microtubule network and DR5-mediated apoptosis.

**Figure 8:**
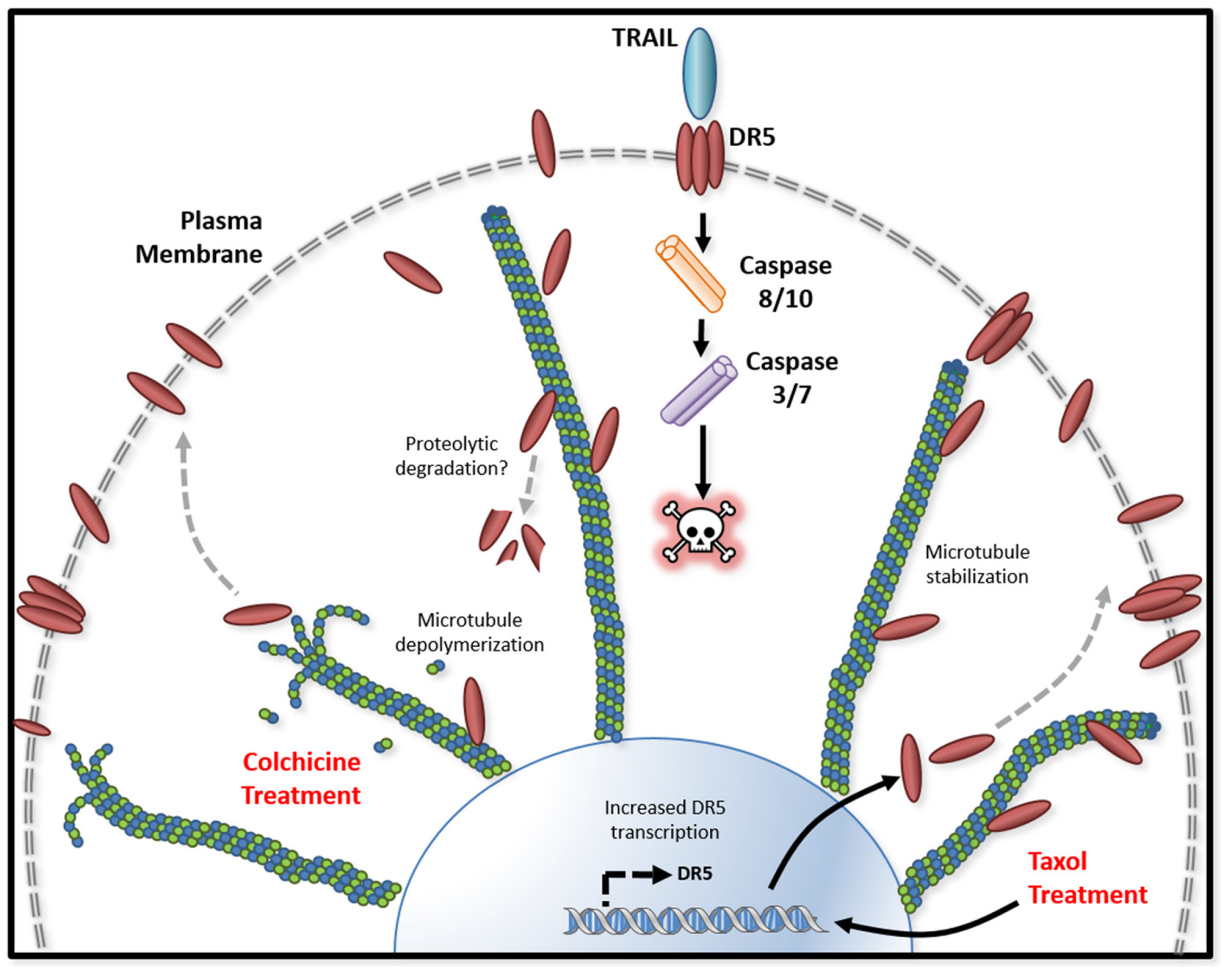
Tubulin/microtubule regulation of DR5 mediated apoptosis Tubulin and DR5 are found to form stable complexes which appears to promote DR5 protein degradation via undefined pathways. Pharmacological disruption of tubulin network using either depolymerizing (e.g., colchicine) or stabilizing (e.g. taxol) agent increases DR5 total and surface expression. In both cases, the resultant cells are sensitized to TRAIL induced apoptosis. Our data suggest distinct mechanisms: 1) Tubulin depolymerization appears to lower affinity to DR5 which results in release and stabilization of DR5 protein, and 2) Tubulin stabilization shows little or effect on DR5-tubulin complex formation, but it may act via inducing DR5 transcriptional expression [21]. In either case, the upregulated DR5 protein levels may be responsible for the increased surface expression and subsequent apoptosis in response to TRAIL treatment.

Tubulin proteins are essential components of microtubules that are featured by cylindrical structures being assembled from α- and β-tubulin heterodimers. The tubulin/microtubule network undergoes dynamic rearrangement in the course of apoptotic execution [13]. In the present study, we found that tubulin physically interacts with DR5 in cancer cells (Figure 1 & 2). Using immunoprecipitation, we identified stable tubulin-DR5 immunocomplexes from cells transfected with Strep-DR5 as well as cells expressing considerable levels of endogenous DR5 protein. Pharmacological disruption of tubulin network i.e. depolymerization or stabilization was found to upregulate DR5 total protein expression and its presentation at the cell surface of targeted cells. In both cases, the resultant cells were sensitized to TRAIL induced apoptosis (Figure 3, 5 & 6). Our data suggest distinct mechanisms: 1) Tubulin depolymerization appears to lower affinity to DR5 which results in release and stabilization of DR5 protein, and 2) Tubulin stabilization shows little or effect on DR5-tubulin complex formation, but it may act via inducing DR5 transcriptional expression [21]. In either case, the upregulated DR5 protein levels may be responsible for the increased surface expression and subsequent apoptosis in response to TRAIL treatment (illustrated in Figure 8). A body of evidence links tubulin to ubiquitin-proteasome protein degradation pathways [20, 24, 25]. For instance, Skp1 and Cul1 (the essential components of SCF E3 ligase complex) and 20S proteasome were found to be localized in the microtubule organizing complex (MTOC) [26]. Tubulin was also shown to act as a scaffold for E3 ubiquitin ligase to facilitate the degradation of Gli2 (a hedgehog signaling mediator) [23]. It is possible that tubulin targets DR5 protein for ubiquitin-proteasome degradation, but the precise mechanisms remain unclear. Interestingly, H460 cells underwent spontaneous apoptosis following tubulin inhibition in the absence of TRAIL ligand (Figure 4). This might be due to the overexpression of DR5 on the cell surface, which was shown to cluster and trigger caspase activation in a ligand-independent manner [27].

Consistent with our data, taxol has been shown to enhance TRAIL killing activity in several cancer cell lines [1, 16-19]. Disrupting the dynamics of tubulin/microtubule formation changes the motility of proteins along the network [28]. While stabilization of the tubulin network did not impact the affinity of DR5 to tubulin, there was a marked increase in the total DR5 protein levels (Figure 3 & 4). Tubulin-stabilizing agents are able to induce DR5 mRNA expression (Figure 8), increasing protein availability through p53 dependent or independent mechanisms [21]. p53 interacts with the microtubule network and its trafficking is impacted upon treatment with microtubule-targeted agents, leading to accumulation within the nucleus [28]. p53 is known to transcriptionally regulate DR5, therefore may contribute to the increase in DR5 total expression upon taxol treatment [27, 29]. Further studies are necessary to understand how p53 mutation status impacts DR5-tubulin interactions.

These data provide a novel mechanism by which therapeutic agents targeting tubulin/microtubules kill cancer cells. Our findings suggest that these drugs may function at least partly through upregulation of DR5 expression, leading to DR5 self-clustering and spontaneous caspase activation in targeted cells. Importantly, we showed synergistic effects of TRAIL and tubulin inhibitors to kill cancer cells. Such combinations may be able to overcome tumor resistance mechanisms, leading to better clinical outcomes in cancer patients.

## MATERIALS AND METHODS

### Cell lines and reagents

Human cancer cell lines MDA-MB-231 (MB-231), H460, HCT116, and 293T were purchased from the American Type Culture Collection (ATCC) and were cultured per the vendor’s protocols at 37°C in a humidified atmosphere with 5% CO_2_. The cell line authentication was proved by growth rate, morphology, isoenzymology, short tandem repeat profiling, and mycoplasma testing (https://www.atcc.org/). Monoclonal antibodies specific to DR5 (Catalog #8074), Phospho-Histone H3 (ser10) (3377), β-tubulin (2128), Poly ADP ribose polymerase (PARP) (9542), caspase 8 (9746), and caspase 3 (9662) were from Cell Signaling Technology (Danvers, MA). Antibody to GAPDH (NB300-328) was from Novus Biologicals (Littleton, CO). Horseradish peroxidase (HRP)–conjugated goat anti-rabbit IgG-HRP (sc-2054) and anti-mouse IgG1-HRP (sc-2969) were from Santa Cruz Biotechnology (Dallas, TX). Anti-DR5 monoclonal antibody (CDM237) and IgG2b isotope control (CSI11889) used in co-immunoprecipitation (co-IP) assays were from Cell Sciences (Newburyport, MA). Antibodies against beta-tubulin (T0198) and α-tubulin (T6199), cycloheximide (CHX; protein synthesis inhibitor) (C4859), poly-L-lysine hydrobromide (P9155), paclitaxel (T1912), and colchicine (C9754) were from Sigma-Aldrich Corporation (St. Louis, MO). Anti-DR5 antibody used for immunocytochemistry was purchased from ProSci Inc (Poway, CA). Goat anti-mouse-AF488 (A10667) and goat anti-rabbit-AF594 (A11012) fluorescence-conjugated secondary antibodies were from Life Technologies (Carlsbad, CA). DAPI-slow fade mounting solution (H-1200) was purchased from Vector Laboratories. Twin-Strep Purification kit (2-1121-011) was purchased from IBA Solutions For Life Sciences (Germany). Pierce Silver Stain for Mass Spectrometry kit (24600) and mouse IgG1 isotype control (MA5-14453) were from ThermoFisher Scientific (Rockville, MD). Recombinant human TRAIL/TNFSF10 (rhTRAIL) (375-TEC), phycoerythrin (PE)-conjugated monoclonal antibody DR5 (FAB6311P) and its corresponding IgG2b (IC0041P) isotope controls were from R&D Systems (Minneapolis, MN). The synthetic small interference RNA (siRNA) oligos specific to tubulin (L-013150-00-0005) and a scramble siRNA (D-001810-10-20) were purchased from Dharmacon. Transfections of siRNAs and cDNA plasmids were performed using Lipofectamine RNAiMAX Reagent from ThermoFisher.

### Affinity purification

Strep-tag affinity purification was conducted using one-Strep starter Kit (IBA Solutions For Life Sciences) per the vendor’s protocol [24]. Briefly, 293T cells were transfected with a cDNA plasmid (pCMV6-AC-Strep-DR5L) encoding Strep-DR5 fusion protein, using Lipofectamine 2000 reagent (ThermoFisher Scientific). Cells transfected with pCMV6-AC empty plasmid were used as a control. At 15 h post-transfection, cells were lysed in RIPA buffer (Sigma) supplemented with protease inhibitors and phosphatase inhibitors (Roche). After centrifugation at 14,000 rpm for 30 min at 4°C, supernatants were collected, and protein concentration was measured with BCA kit. After centrifugation, supernatants were applied onto pre-equilibrated Strep-Tactin columns. The bound proteins were eluted and resolved onto SDS-PAGE and stained using Pierce Silver Stain for Mass Spectrometry kit (ThermoFisher Scientific). analyzed by mass spectrometry. The selected protein bands were retrieved, and subjected to in-gel trypsin digestion, which was followed by tandem Mass Spectrometry (MS) analysis for protein identification as described previously [25]. Isolation of endogenous immunocomplexes was performed using anti-DR5 antibody as a bait following a similar procedure with equal amounts of input total protein, as determined by BCA.

### Cell viability assays

Cell viability was determined using CellTiter 96 AQ_ueous_ One Solution Cell Proliferation Assay kit from Promega (Madison, WI). Briefly, 20 μl of CellTiter 96 AQ_ueous_ One Solution reagent was added into each well of the 96-well plate containing 100 μl of culture medium. After incubation for 1-4 hours, absorbance was recorded at 490 nm using a 96-well plate reader (SpectraMax Plus 384, Molecular Devices). For colchicine and TRAIL synergy analysis, cells were treated with a single drug dose range of colchicine (50-200 ng/mL) or TRAIL (5-20 ng/mL) as well as in combination.

### Flow cytometry

DR5 expression on the cell surface was analyzed using flow cytometry. Cells at 70-80% confluence were washed with 1× PBS and harvested by incubation with enzyme-free dissociation buffer. Harvested cells were counted using Cellometer and spun down at 1,350 rpm. The cell pellets were re-suspended in a blocking buffer (5% normal goat serum and 1% bovine serum albumin in PBS) to reach 5 × 10^6^ cells/mL. After 20 min incubation on ice, 30 μl of cell suspension was mixed with 10 μl of 10 μg/ml anti-DR5-PE or corresponding IgG2b-PE as control and incubated for 45 min on ice in the dark. Cells were washed with PBS and re-suspended in 500 μl of PBS and analyzed on an Accuri C6 flow cytometer (BD Biosciences).

### Immunoblotting

Whole cell lysates were prepared in RIPA buffer (Sigma-Aldrich). Protein concentrations were measured using BCA Protein Assay Kit (ThermoFisher Scientific). Equal amounts of total proteins were resolved onto SDS-PAGE using the 4-12% NuPAGE Bis-Tris gels (ThermoFisher Scientific) and transferred to PVDF membranes. Membranes were blocked, washed and incubated with primary antibody followed by secondary antibody and visualized by ECL reagents. Densitometry analysis was performed with the LAS-4000 Luminescent Image Analyzer (Fujifilm). Bands density was quantified with Image J software (http://imagej.nih.gov).

### Mass spectrometry

Silver-stained protein bands were retrieved from SDS-PAGE gels. Proteins were in-gel digested with trypsin in 25 mM ammonium bicarbonate overnight at 37 °C, followed by extraction of peptides with 60% acetonitrile in 0.1% formic acid. Samples were dried in a Savant SpeedVac concentrator (Thermo Fisher Scientific), and then dissolved in 3% acetonitrile in 0.1% formic acid. The peptides were separated on a 43-mm HPLC C18 ProtID-Chip (Agilent Technologies), followed by data-dependent auto-MS/MS on an nano-electrospray-ionization 6520 Q-TOF tandem MS spectrometer (Agilent Technologies). Protein identifications were achieved by searching Swiss-Prot Homo sapiens protein database using Spectrum Mill MS Proteomics Workbench software (version B.04.00; Agilent Technologies) with a global false discovery rate of 1%.

### Confocal microscopy

Immunocytochemistry and confocal microscopy visualization was performed using a Zeiss LSM880 (Zeiss). Glass microscopy slides were coated with Poly-L-lysine before cells were seeded overnight. Cells were fixed with 4% paraformaldehyde and permeabilized with 0.01% Triton-X. Slides were blocked with 3% bovine serum albumin in PBS before labeling. Immunolabeling for DR5 and alpha tubulin was visualized with fluorescence-tagged antibodies and counterstained with DAPI. Images were obtained at 40x magnification and analyzed using Zen software (Zeiss). Negative controls were unlabeled samples and secondary antibody only stained samples.

### Statistical analysis

Statistical analysis was performed using GraphPad Prism 6 (GraphPad Software). Statistical comparisons for cell viability, surface protein and total protein expression were determined using one-way ANOVA and un-paired *t*-test with a Welch’s correction. All assays were completed with an N≥ 3. Statistical significance is shown as either ^*^p<0.05 or ^**^p<0.01 as indicated in the results. The effects of drug combination were determined using the CompuSyn software (http://www.combosyn.com). Combination Index (CI) values were then calculated based on cell viability data. CI values of <1, =1 and >1 represent synergism, additive effect and antagonism, respectively.

## SUPPLEMENTARY MATERIALS FIGURE


