# Supplementary material for: Tubulin couples death receptor 5 to regulate apoptosis
Source: Oncotarget. 2018 Dec 4;9(95):36804–15. doi: 10.18632/oncotarget.26407 (PMC6298406; doi:10.18632/oncotarget.26407)
Supplement: Supplementary file 1 [file oncotarget-09-36804-s001.pdf]

Tubulin couples death receptor 5 to regulate apoptosis

SUPPLEMENTARY MATERIALS

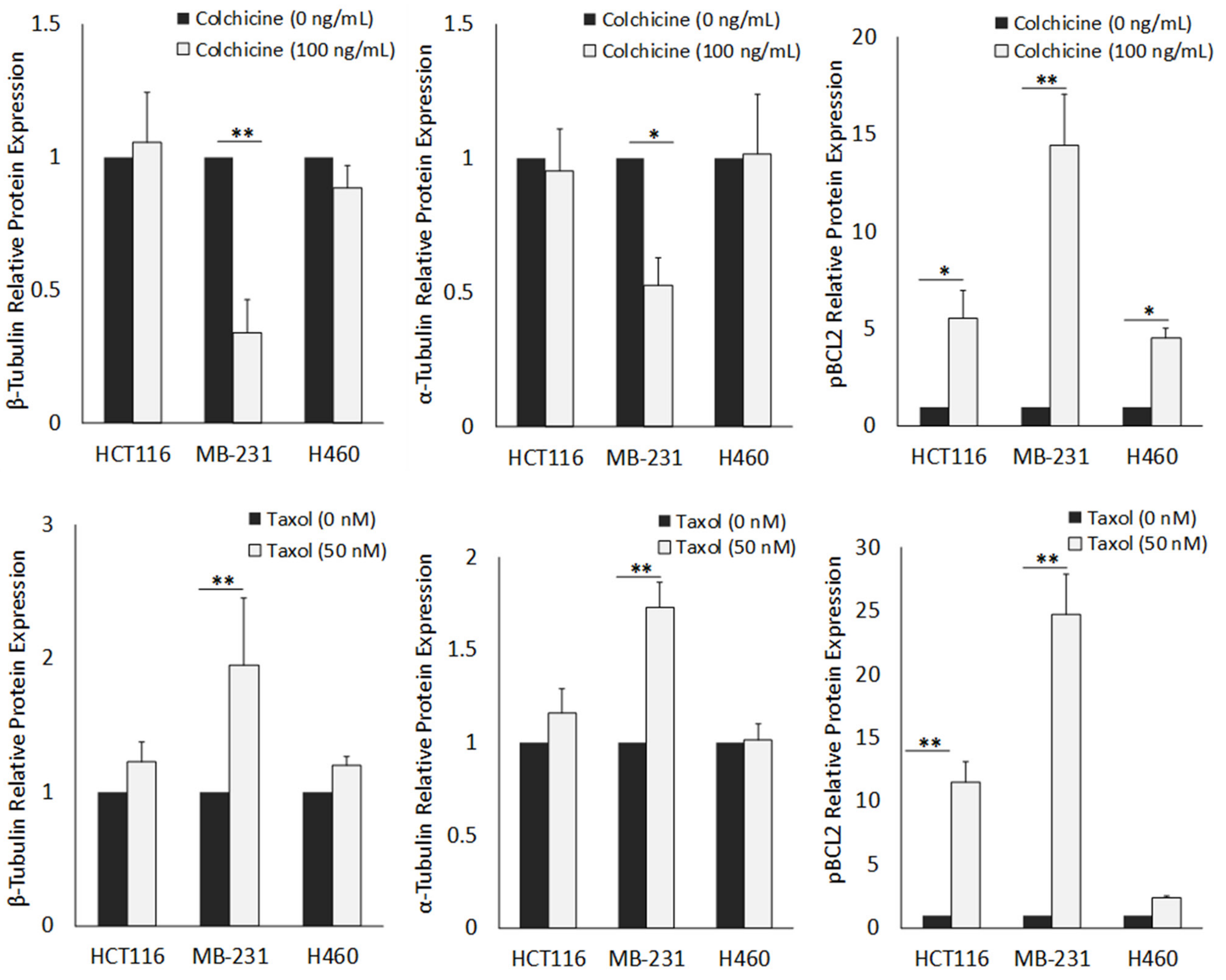

Supplementary Figure 1: Relative protein levels were quantified from the blots of HCT116, MB-231, and H460 cells treated with colchicine at 100 ng/mL or taxol at 50 nM for 16 hours. Data shown is relative to GAPDH internal control and non-treated samples. Values are means ± SD, $^{*}p<0.05$, $^{**}p<0.01$, N=3.
